# p38 Mitogen-Activated Protein Kinase Pathway Regulates Genes during Proliferation and Differentiation in Oligodendrocytes

**DOI:** 10.1371/journal.pone.0145843

**Published:** 2015-12-29

**Authors:** Jeffery D. Haines, Debra L. Fulton, Stephane Richard, Guillermina Almazan

**Affiliations:** 1 Department of Pharmacology and Therapeutics, McGill University, 3655 Sir William Osler Promenade, Montreal, Quebec, Canada, H3G 1Y6; 2 Department of Neurology and Neurosurgery, Montreal Neurological Institute and Hospital, McGill University, 3801 University St, Montreal, Quebec, Canada, H3A 2B4; 3 Terry Fox Molecular Oncology Group, Bloomfield Center for Research on Aging, Lady Davis Institute for Medical Research, and Departments of Oncology and Medicine, McGill University, Montreal, Quebec, Canada, H3T 1E2; George Mason University, UNITED STATES

## Abstract

We have previously shown that p38 mitogen-activated protein kinase (p38 MAPK) is important for oligodendrocyte (OLG) differentiation and myelination. However, the precise cellular mechanisms by which p38 regulates OLG differentiation remain largely unknown. To determine whether p38 functions in part through transcriptional events in regulating OLG identity, we performed microarray analysis on differentiating oligodendrocyte progenitors (OLPs) treated with a p38 inhibitor. Consistent with a role in OLG differentiation, pharmacological inhibition of p38 down-regulated the transcription of genes that are involved in myelin biogenesis, transcriptional control and cell cycle. Proliferation assays showed that OLPs treated with the p38 inhibitor retained a proliferative capacity which could be induced upon application of mitogens demonstrating that after two days of p38-inhibition OLGs remained poised to continue mitosis. Together, our results suggest that the p38 pathway regulates gene transcription which can coordinate OLG differentiation. Our microarray dataset will provide a useful resource for future studies investigating the molecular mechanisms by which p38 regulates oligodendrocyte differentiation and myelination.

## Introduction

Oligodendrocyte (OLG) maturation involves a complex interplay of cell cycle regulators, transcriptional activators and repressors that drive terminal differentiation (extensively reviewed by [1–3]. We have previously shown that p38 mitogen-activated protein kinase (MAPK) regulates OLG differentiation and central nervous system (CNS) myelination [4, 5]. Pharmacological inhibition of p38 in oligodendrocyte progenitors (OLPs) prevents the accumulation of myelin-specific mRNAs and proteins such as myelin basic protein (MBP) and myelin-associated glycoprotein (MAG) [5]. p38 MAPK has also shown to direct myelin-specific gene expression through the differential regulation of myelin-promoter activities [6]. Furthermore, *in vitro* genetic knock-down of p38α reduces MAG levels, and galactosylceramide (GalC) staining in OLG membrane sheets. Our previous studies also revealed that the downstream p38 MAPK effector, MK2, is a component of the signaling pathway that promotes OLG differentiation [7]. However, the mechanisms by which p38 MAPK and MK2 regulate OLG differentiation are unknown. Complementing our in vitro work, a recent study has shown that OLG progenitors derived from p38α conditional knockout mice also failed to differentiate in culture. Moreover, electron microscopic analysis showed that the ultrastructure of myelin bundles was impaired and the onset of myelination was delayed in the corpus callosum in p38α knockout mice [8].

To further elucidate the mechanisms by which p38 MAPK signaling regulates OLG differentiation, we used rat whole genome microarray profiling on oligodendrocyte progenitors (OLPs) treated with the p38α/β isoform inhibitor, PD169316. In addition to the anticipated alterations in myelin gene expression, we identified novel gene targets regulated by the p38 pathway, including transcripts encoding proteins that are involved in vesicular transport, transcription factors previously shown to regulate genes in OLGs, and cell cycle regulators. We validated differential expression of several associated gene transcripts by qPCR. Subsequent proliferation assays indicate that OLPs treated with p38 inhibitors are poised in an active cell cycle state before S-phase. Our results suggest that the p38 pathway regulates genes that function to direct OLG identity through cell cycle and eventual arrest to promote terminal differentiation.

## Materials and Methods

### Reagents and supplies

Ham's F12 medium, PBS, 7.5% BSA fraction V, and penicillin/streptomycin were purchased from Invitrogen (Burlington, ON, Canada). Fetal calf serum and Dulbecco’s Modified Eagle’s Medium (DMEM) were from Wisent Inc (St-Bruno, QC); PDGF-AA and bFGF from PeproTech (Rocky Hill, NJ). PD169316 was from EMD Chemicals (San Diego, CA). Poly-D-lysine, poly-L-ornithine, human transferrin, insulin, HEPES, Triton-X-100, DTT were from Sigma-Aldrich. Western blotting reagents from GE Healthcare Life Sciences (Baie d’Urfe, QC); A2B5 mouse monoclonal antibody from American Type Culture Collection; rabbit polyclonal Ki67 conjugated with FITC from Abcam (Toronto, ON); mouse monoclonal anti-p27kip1 (BD Biosciences, Mississauga, ON); rabbit polyclonal p57 (H-91) from Santa Cruz; rabbit monoclonal phopho-CDC2 (TYR15) from Cell Signaling Technology (Danvers, MA); HRP-, FITC-, or Texas Red-conjugated secondary antibodies from Southern Biotechnology, Jackson Immunoresearch Laboratories (Cedarlane, Hornby, ON), BIO-RAD Canada (Mississauga, ON) or Invitrogen (Burlington, ON); Hoechst nuclear stain from Molecular Probes Inc. (Eugene, OR). The O4 antibody was a gift (Sommer and Schachner 1981). All other reagents were from Fisher Scientific (Whitby, ON), or VWR (Mont-Royal, QC)

### Cell cultures

Primary cultures of oligodendrocyte progenitors (OLPs) were prepared from the brains of newborn Sprague-Dawley rats as described previously (McCarthy and de Vellis 1980; Almazan, Afar et al. 1993). All experiments were approved by the McGill Faculty of Medicine Animal Care Committee (permit number 4373) in accordance with Canadian Council on Animal Care guidelines. OLPs were plated on poly-D-lysine (PDL)-coated culture dishes and grown in serum free media (SFM) consisting of a DMEM-F12 mixture (1:1), 10 mM HEPES, 0.1% bovine serum albumin, 25 mg/mL human transferrin, 30 nM triiodothyronine, 20 nM hydrocortisone, 20 nM progesterone, 10 nM biotin, 5 mg/mL insulin, 16 mg/mL putrescine, 30 nM selenium and 2.5 ng/mL each of PDGF-AA and bFGF. The OLPs were changed with media that included mitogens every 2d to maintain the cells in a proliferative state. OLPs spontaneously differentiate upon removal of mitogens. Cultures were characterized immunocytochemically with cell-type-specific antibodies as previously reported (Cohen and Almazan 1994; Radhakrishna and Almazan 1994). On day 0 of differentiation, more than 95% of the cells are positive for gangliosides detected with monoclonal antibody A_2_B_5_, a marker for OLPs in culture while less than 5% were GalC-positive OLGs, GFAP-positive astrocytes or complement type-3-positive microglia. Cells start to express surface sulfatides, recognize with the monoclonal antibody O4 on day 1 following growth factor removal, GalC and MAG (myelin-associated protein) on day 2–3 and MBP (myelin basic protein) on days 3–4. The culture media was changed every 2d, and PD169316 was used at 5.0 μM for all experiments, unless otherwise indicated. An equivalent DMSO concentration treatment was used for the control.

### RNA extraction

Total RNA was extracted from OLGs (~500,000 cells) differentiated in the absence or presence of 5 μM PD169316 using the Qiagen RNeasy kit (Qiagen, Mississauga, ON, Canada). Genomic DNA was eliminated using an on-column DNase digest (DNase set, Qiagen). The RNA was divided into separate aliquots for microarray analysis and quantitative PCR validations. RNA quality was assessed by Génome Québec Innovation Centre (GQIC) using an Agilent RNA BioAnalyzer (Agilent Technologies, Santa Clara, CA), followed by a Qiagen RNA clean-up column, and quantification using a Nanodrop ND-1000 spectrophotometer (Nanodrop Technologies, Wilmington, DE).

### Illumina microarray

Gene expression was determined in control and PD169316-treated OLGs using Illumina Rat Whole Genome microarrays in collaboration with GQIC. Complimentary RNA (cRNA) was prepared from 250 ng of total RNA using the Ambion TotalPrep RNA Amplification kit according to the manufacturer's protocol. cRNA (750 ng/array) generated from this kit was hybridized to Rat Illumina Whole Genome Microarrays (RatRef-12 array) according to Illumina's protocol. The microarray data is available in the NCBI Gene Expression Omnibus (Reference Number: 15960825). All data are MIAME compliant, and the raw data have been deposited in a MIAME compliant database, as detailed on the MGED Society website http://www.mged.org/Workgroups/MIAME/miame.html.

### FlexArray analysis

The GQIC FlexArray analysis software provides ratio data for treatment values compared to control samples for each gene, a t-statistic and the associated p value. The data was transformed to fold-change using -1/x transformation for ratio values below 1.0. Values above 1.0 were not transformed. There were 3 samples in each treatment group. Genes with a fold change value +/- 1.25, with a p value less than 0.05 were considered to be statistically differentially expressed over control. The FlexAray EB (Wright and Simon) statistical correction was applied to the p-values. Tabulated microarray results are available in the online supporting information.

### Functional Determination using UniProt and literature searches

The "Core Analysis" function included in the Ingenuity Pathway Analysis (Ingenuity Systems, Redwood City, CA, USA) was used to interpret the rat whole genome microarray data in the context of biological processes, pathways and networks that were modulated by PD169316-treatment. The genes were identified through their unique NCBI Accession number. The biological function of the most up-regulated and down-regulated genes was determined using UniProt Gene Ontology annotations (http://www.uniprot.org/), or through functional annotations provided in published literature available through PubMed (http://www.ncbi.nlm.nih.gov/pubmed/). The complete table of up- and down-regulated genes classified according to their biological function is available in S1 Table.

### Quantitative PCR

One microgram of DNaseI-treated RNA was reverse-transcribed using the AMV reverse transcriptase (Roche Diagnostics, Laval, Quebec). When possible, primers were designed to span exon-exon junctions (**S2 Table**). One μL of the total cDNA sample was analyzed per reaction, using the 96 well-block Roche LightCycler 480, and SYBR Green master mix (SABiosciences, Frederick, MD, USA). PCR amplifications were performed as follows: heat inactivation (10 min, 95°C); followed by 35–45 cycles of 94°C, 15 s; 59°C, 30 s; 72°C, 20 s. PCR products were detected by fluorescence at the end of the extension step, and melting curves were analyzed by monitoring the continuous decrease in fluorescence of the SYBR Green signal. PCR products were verified for a single amplification product using melting curve analysis, and the molecular weight of each product was confirmed using PAGE. The fold change in mRNA levels was determined using advanced relative quantification (Pfaffl) method available in the Roche LightCycler 480 software, and data were normalization with 28S rRNA expression levels.

### Proliferation assays

Cells were grown to an approximate density of 1.5 × 10^5^ cells/cm^2^ in 24-well dishes. OLPs were treated with an increasing dose (1–7.5 μM) of PD169316 for 24 or 48 hrs in SFM without mitogens, and then incubated with 1 μCi/mL ^3^H-thymidine for an additional 24 hr. In experiments where mitogens were applied after PD169316 treatment, 2.5 ng/mL PDGF-AA and bFGF were added at the same time as the ^3^H-thymidine and incubated for 24 hr, as above. Following the ^3^H-thymidine incorporation period, the medium was aspirated and cultures were rinsed three times with 5% ice-cold trichloroacetic acid and solubilized in 0.2 N NaOH and 0.1% Triton-X-100. Aliquots were mixed with Ecolite liquid scintillation counting fluid and emissions were recorded using a β -counter.

### Time course immunocytochemistry and in vitro quantifications

Primary OPCs were plated on PDL-coated glass coverslips in 24-well plates. When the cells reached 75–80% confluency (denoted Day-0) the cells were treated with 5μM PD169316 in SFM (treatment) and comparable volume of DMSO in SFM (control). The treatment was maintained up to 96 hours then the cells were treated with 20 ng/mL each of PDGF-AA and bFGF. Coverslips were collected 24 hours later and stained with A2B5, O4, and Ki67 antibodies and Hoechst nuclear staining. A2B5 + O4, and Ki67 immunoreactive cells were counted on a minimum of 12 independent fields (2 fields/3 coverslips/treatment x 2 experiments) in photomicrographs captured with a 20X objective. Labeled cells in photomicrographs were identified both manually and via automated ImageJ analyses and enumerated with the aid of ImageJ cell counting software tools.

## Results

### p38 MAPK regulates diverse cellular targets in OLGs

**Fig 1:**
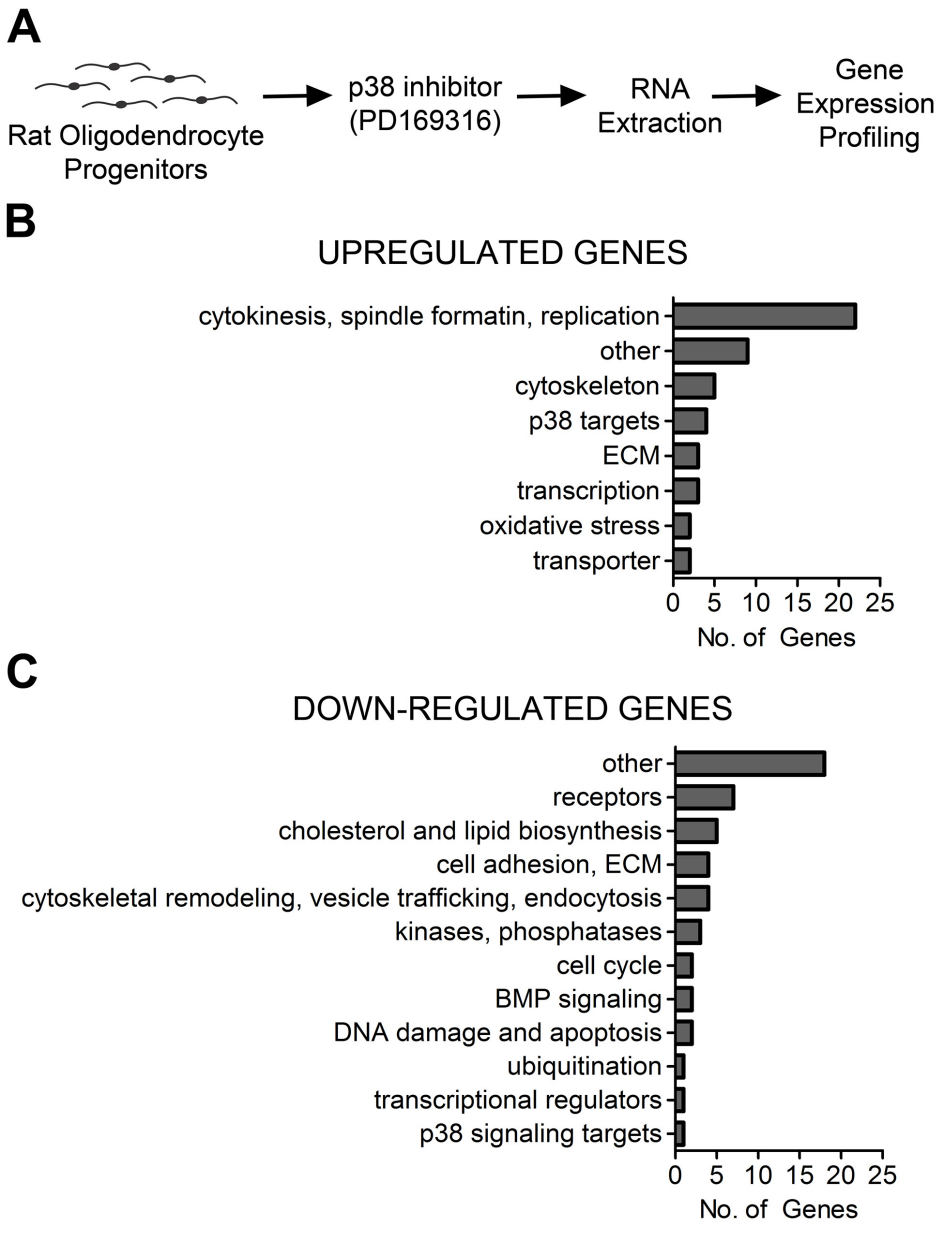
Gene expression profiling of oligodendrocyte progenitors treated with the p38 MAPK inhibitor, PD169316, reveals gene targets with diverse cellular functions. (A) Schematic diagram of the microarray analysis performed on rat oligodendrocyte (OL) progenitors treated for 1d with the p38 inhibitor, PD169316. Groups of genes were classified by biological function using UniProt and Gene Ontology functional analysis. (B) Functional classes of up-regulated genes included those involved in cytokinesis, spindle formation, replication, cytoskeleton and vesicular transport, p38 target genes, extracellular matrix (ECM), transcription and oxidative stress. (C) down-regulated gene transcript classes included receptors, ligands and transporters, cholesterol and lipid biosynthesis, extracellular matrix (ECM), cytoskeletal remodeling and vesicle trafficking, kinases, phosphatases, cell cycle, bone morphogenetic (BMP) signaling, DNA damage and apoptosis, ubiquitination, transcriptional regulators, and p38 signaling targets.

To further understand how p38 regulates OLG differentiation, we performed an Illumina microarray analysis on primary rat OLPs differentiated in the presence of the p38α/β isoform-specific inhibitor PD169316 to identify gene targets regulated by this signaling pathway. The microarray data was analyzed to determine transcript expression ratio differences as compared to DMSO-treated controls (Fig 1A) and transformed to fold change. Genes that were up- or down-regulated 1.25 fold with a statistical p-value less than or equal to 0.05 were considered to be significantly differentially expressed from control. We classified the fifty most up- and down-regulated genes that were differentially expressed at 48 hrs (2d) after PD169316 treatment according to their biological functions using the Gene Ontology (GO) annotations [9] found in the UniProt database [10] or using literature-derived functional annotations. Up-regulated classes included genes involved in cytokinesis, centromere and spindle formation, replication and cell cycle progression. Smaller subsets of genes up-regulated by PD169316 treatment included amino acid transporters, cytoskeletal proteins, vesicular transporters, extracellular matrix molecules, and genes involved in oxidative stress. Down-regulated gene transcripts encoded proteins belonging to bone morphogenetic signaling pathways, chondroitin sulfate proteoglycans, and cytoskeletal/vesicle trafficking proteins. A prominent set of down-regulated genes remained unclassified with no known function (Fig 1B and 1C and S1 Table).

### Levels of myelin gene transcripts were correctly regulated during OLG differentiation

To confirm that the microarray gene expression profiles aligned with previously known gene expression following OLG differentiation, we analyzed transcript levels of typical immature and mature cell-stage specific markers. OLPs continue to proliferate when cultured in the presence of the mitogens PDGF-AA and bFGF and differentiate upon their removal. Consistent with a decreased mitogenic effect upon differentiation, lower expression levels were seen for immature cell-stage markers such as the PDGF receptor α (*Pdgfra*), *nestin*, and the early specification marker of the Nkx homeobox gene class family, *Nkx2*.*2*. In addition, we observed an upregulation of myelin-specific genes such as *Mag*, *Mbp*, myelin oligodendrocyte glycoprotein (*Mog*), and myelin and lymphocyte protein (*Mal*). The Nkx homeobox gene *Nkx6*.*2* expression levels were also increased, consistent with its role in OLG differentiation [11]. The expression of another pro-myelin gene activator *Hdac11*, which promotes the expression of *Mbp* and *Plp* during OLP maturation, was also elevated during OLG differentiation [12] (Table 1).

**Table 1:** Myelin-specific and other pro-myelin gene transcripts upregulated and/or downregulated during OLG differentiation. OLGs were differentiated by removal of PDGF-AA and bFGF for 2d.

Gene ID	Gene Name	Accession Number	Fold Change	p value
*Mal*	Myelin and lymphocyte protein	NM_012798.1	12.59	1.065E-05
*Mog*	myelin associated glycoprotein	NM_022668.1	10.33	9.30E-07
*Mbp*	myelin basic protein, transcript variant 3	NM_001025293.1	6.58	3.75E-06
*Nkx6*.*2*	NK6 transcription factor related, locus 2	XM_219447.4	4.15	1.18E-04
*Hdac11*	histone deacetylase 11	XM_001073226.1	4.09	3.81E-06
*Mag*	myelin-associated glycoprotein	NM_017190.4	3.24	4.71E-07
*Cgt/Ugt8*	UDP galactosyltransferase 8	NM_019276.2	1.79	3.62E-05
*Pdgfra*	platelet derived growth factor receptor, alpha polypeptide, transcript variant 2	XM_001067631.1	-2.34	6.65E-06
*Nkx2*.*2*	NK2 transcription factor related, locus 2 (Drosophila)	XM_001056116.1	-4.68	4.84E-06

### p38 inhibition decreases levels of transcripts encoding myelin-specific genes

**Fig 2:**
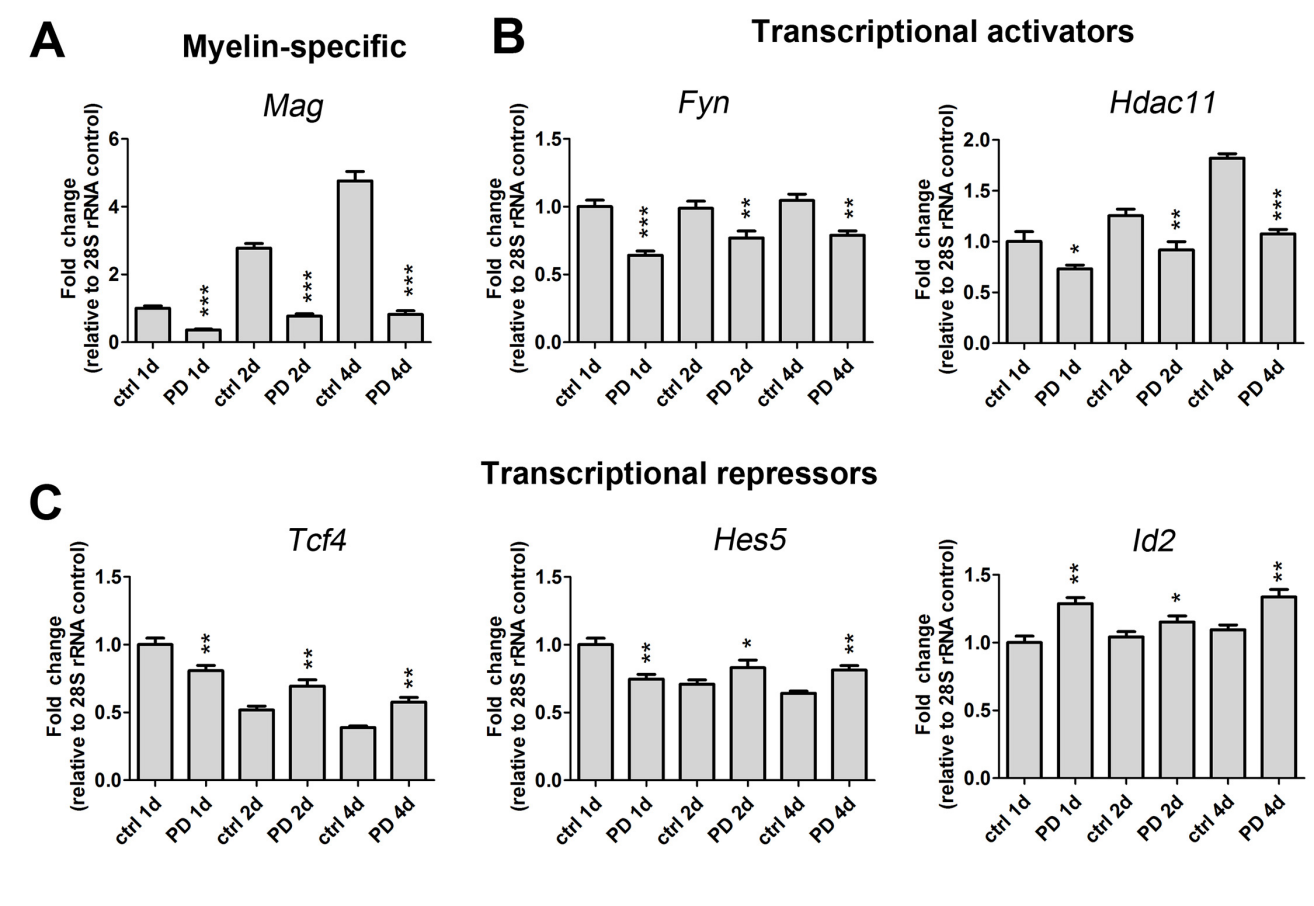
p38 MAPK regulates the expression of myelin gene transcripts and myelin gene activators and repressors that control oligodendrocyte identity. Gene transcript expression levels of (A) myelin specific (*Mag*), (B) transcriptional activators (*Fyn*, *Hdac11*) and (C) transcriptional repressors (*Tcf4*, *Hes5*, *Id2*) are altered after PD169316 treatment as determined by qRT-PCR. OLPs were treated with 5 μM PD169316 for 1d, 2d or 4d and RNA was harvested, reverse transcribed and analyzed by qRT-PCR. All gene transcripts were normalized to ctrl at 1d all relative to 28S rRNA. Statistical differences were determined using independent t-tests with Bonferroni’s correction (*p < 0.05, **p< 0.01, ***p< 0.001 vs same day ctrl).

We next set out to determine whether specific gene expression changes marked the arrest of OLG lineage progression after treatment with a p38 inhibitor. We previously found that PD169316 treatment decreases levels of myelin-specific gene transcripts, including *Mbp*, *Mag*, myelin oligodendrocyte basic protein (*Mobp*), and UDP-galactose ceramide galactosyltransferase (*Ugt8*) [5]. In agreement with our previous findings, the Illumina microarray data revealed significantly decreased gene expression levels for *Mbp*, *Ugt8* and *Mag* after 24 h (1d) treatment with PD169316. Correspondingly, we also observed decreases in oligodendrocyte myelin glycoprotein (*Omgp*). Corroborating our microarray analyses, we detected a large down-regulation of *Mag* by qRT-PCR analysis at three time points during differentiation in the presence of PD169316 (Fig 2A). We also detected decreases in non-OLG specific genes including squalene epoxidase, an enzyme involved in a key step in the synthesis of cholesterol that is a necessary component of myelin membrane formation [13]. *Fyn* a Src-like tyrosine kinase family member that is important for OLG differentiation and CNS myelination [14], was also down-regulated (Table 2 and Fig 2B). Further, PD169316 treatment decreased the transcription of genes encoding *Nkx6*.*2*, *Hdac11*, *Sox8* and zinc-finger protein 488 (*Zfp488*), all of which are implicated in activating myelin gene transcription. We employed qRT-PCR analysis to validate the expression level decrease in *Hdac11* and confirmed a decrease at three different time points of differentiation (Fig 2B). Additionally, we observed decreased mRNA levels of thyroid hormone receptor, *Thra*, which binds T3 to activate an intracellular timer controlling OLP differentiation [15].

**Table 2:** Myelin genes and transcriptional activators are decreased by a 24h treatment of OLPs with 5 mM PD169316.

Gene ID	Gene Name	Accession Number	Fold Change	p value
*Fyn*	fyn proto-oncogene	XM_001062721.1	-1.92	8.44E-09
*Mbp*	myelin basic protein, transcript variant 3	NM_001025293.1	-1.51	6.98E-04
*Sox8*	SRY-box containing gene 8 (predicted)	XM_001060343.1	-1.50	7.26E-07
*Cgt/Ugt8*	UDP galactosyltransferase 8	NM_019276.2	-1.46	7.48E-07
*Sqle*	squalene epoxidase (Sqle)	NM_017136.1	-1.45	1.53E-04
*Hdac11*	histone deacetylase 11	XM_001073226.1	-1.43	1.27E-06
*Thra*	thyroid hormone receptor alpha (Thra), transcript variant TRalpha2	NM_031134.2	-1.43	6.98E-07
*Omgp*	oligodendrocyte-myelin glycoprotein	NM_001005898.2	-1.40	4.55E-06
*Nkx6*.*2*	NK6 transcription factor related, locus 2	XM_219447.4	-1.29	0.40 (NS)
*Zfp488*	zinc finger protein 488	XM_224697.4	-1.29	1.12E-03
*Mag*	myelin-associated glycoprotein	NM_017190.4	-1.22	1.96E-03

NS, non-significant.

### p38 inhibitors increase mRNA levels of OLG transcriptional repressors and markers associated with early OLPs

**Fig 3:**
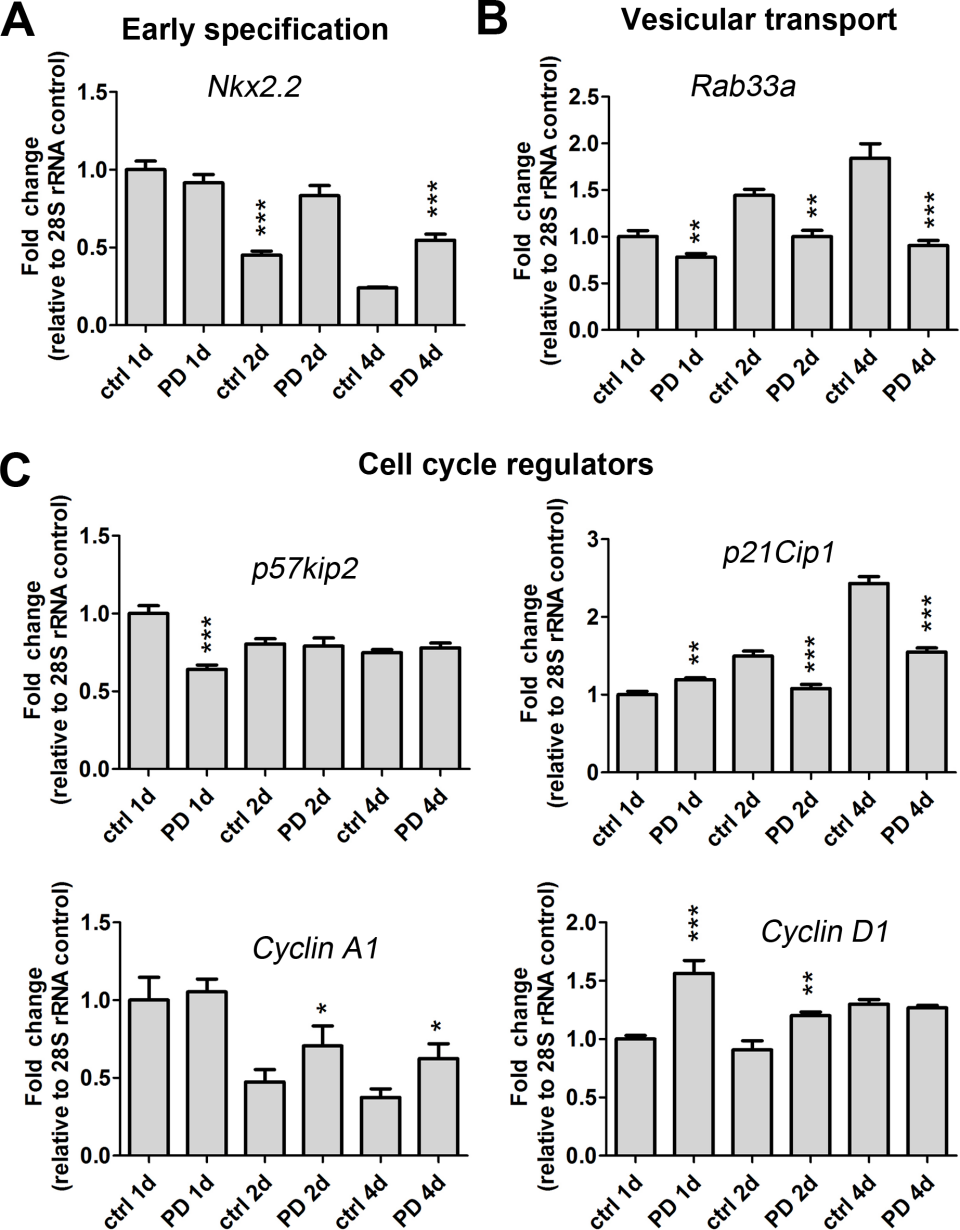
p38 MAPK regulates expression of oligodendrocyte specification genes, vesicular transport regulators and cell cycle regulators. Gene transcript expression levels of early (A) specification markers (*Nkx2*.*2*), (B) vesicular transport (*Rab33a*), and (C) cell cycle regulators (*p57kip2*, *p21Cip1*, *Cyclin A1*, *Cyclin D1*) are altered after PD169316 treatment as determined by qRT-PCR. OLPs were treated with 5 μM PD169316 for 1d, 2d or 4d. All gene transcripts were normalized to ctrl at 1d all relative to 28S rRNA. Statistical differences were determined using independent t-tests with Bonferroni correction (*p< 0.05, **p< 0.01, ***p< 0.001 vs. same day ctrl).

Several transcriptional repressors have been identified that inhibit the progression of OLP maturation and function to regulate the onset of OLG differentiation. These include inhibitors of differentiation (Ids), the Wnt/β-catenin target Tcf4, Sox5/6, Notch and one of its downstream effectors Hes5. The expression levels of these repressors decline with OLG maturation to facilitate the progression of differentiation [16]. In agreement with previously identified roles as inhibitors of differentiation, we observed an up-regulation of *Id1* and *Id2* following PD19316 treatment (Table 3). We also detected up-regulated levels of *Nestin* and *Nkx2*.*2*, which are markers of early OLP development. However, contrary to normal OLP differentiation, the expression level of *Pdgfra* was not affected by PD169316 treatment (S1 Table). While there were no appreciable increases in transcription factors *Notch*, *Hes5* and *Sox6* in our microarray results (S1 Table), we did detect a modest, statistically significant up-regulation at day 2 for *Hes5*, and *Tcf4* by qRT-PCR after p38 inhibition (Fig 2). In agreement with our microarray data, qRT-PCR analyses corroborated up-regulation of the early specification marker *Nkx2*.*2* following p38 inhibition (Fig 3).

**Table 3:** Transcriptional repressors and early OLP markers are upregulated following 24h treatment with 5 mM PD169316.

Gene ID	Gene Name	Accession Number	Fold Change	p value
*Id1*	inhibitor of DNA binding 1	NM_012797.2	1.73	1.33E-06
*Nes*	nestin (Nes)	NM_012987.1	1.46	1.56E-06
*Jund*	Jun D proto-oncogene (Jund)	XM_001070425.1	1.37	3.80E-04
*Id2*	inhibitor of DNA binding 2 (Id2)	NM_013060.2	1.35	2.01E-05
*Nkx2*.*2*	NK2 transcription factor related, locus 2 (Drosophila) (predicted)	XM_001056116.1	1.32	0.32 (NS)
*Sox6*	SRY-box containing gene 6	XM_215016.3	1.22	0.23 (NS)
*Pdgfra*	platelet derived growth factor receptor, alpha polypeptide, transcript variant 2	XM_001067631.1	-1.09	0.22(NS)

### p38 inhibition upregulates the expression of upstream activators of the p38 MAPK pathway

The p38 signaling cascade is initiated by a repertoire of growth stimuli to activate a kinase cascade, which includes a number of MAPK kinase kinases (MKKKs). These enzymes, in turn, activate MKK3 or MKK6 (*Map2k6*) to subsequently activate p38 via phosphorylation of an activation loop domain on p38. Interestingly, we observed an up-regulation of MKK6 following PD169316 treatment suggesting that p38 pathway itself may modulate levels of its upstream activators. In addition, the expression level of PDZ-binding kinase (*Pbk*), an atypical upstream MKK activator of p38, was also significantly elevated following treatment of OLPs with PD169316. PBK has been shown to specifically activate p38 during cell cycle progression and proliferation [17]. Consistent with a role in regulation of its upstream factors, transcript levels of a p38 MKKK gene, *Dlk-1* [18, 19], was also elevated after p38 inhibition (Table 4).

**Table 4:** PD169316 treatment elevates mRNA levels of p38 upstream activators.

Gene ID	Gene Name	Accession Number	Fold Change	p value
*Pbk*	PDZ binding kinase	XM_224300.4	1.93	3.40E-08
*Dlk1*	delta-like 1 homolog (Drosophila) (Dlk1), mRNA.	NM_053744.1	1.88	1.48E-06
*Map2k6 / Mkk6*	mitogen-activated protein kinase kinase 6 (Map2k6)	NM_053703.2	1.53	1.67E-05

### p38 inhibition modulates the expression of cell cycle regulators, early growth response proteins and cytokinesis regulators

The most abundant group (~45%) of up-regulated genes was composed of transcripts that encode proteins involved in cell cycle progression, spindle and centromere formation, and cytokinesis (Fig 1B). Several cyclins (A2, B2, D1), cyclin associated proteins, and cell division proteins were markedly up-regulated in our microarray and qRT-PCR data following treatment of OLPs with PD169316 (Table 5 and Fig 3) [20]. The p38 pathway also regulates cell cycle checkpoints through phosphorylation of p53 [21]. While we found no changes in mRNA levels of *p53* (S1 Table), we did detect decreased levels of p53 target genes including *p21cip1* (Fig 3) and *Gadd45a* (Table 5). We found up-regulated levels for two mitotic checkpoint serine/threonine-protein kinases *Bub*1 (budding uninhibited by benzimidazoles 1 homolog) and a related isoform, *Bub1b*. Moreover, PD169316 treatment increased the transcriptional levels of a large group of kinesins involved in microtubule spindle formation during mitosis (Table 5). An up-regulation of cytoskeletal genes involved in mitotic spindle assembly and chromosome separation were also observed, which included: t*ubulin -b2*, *-b3*, *-b5*, *-b6*, *-g* and *anillin*. Transcript levels for *Rab33a*, a protein involved in vesicular transport were downregulated after PD169316 treatment (Table 5 and Fig 3). Further, transcript expression for p21-activated kinase 7 (*Pak7*), which regulates Rac/Cdc42 GTPases and cytoskeletal dynamics, was decreased after treatment. We found increased mRNAs levels of a set of genes encoding enzymes with roles associated with replication and DNA synthesis, including minichromosome maintenance deficient (MCM), DNA primase and replication factor C. Moreover, the transcription levels of a set of genes involved in spindle body formation and centromere formation were increased after PD169316 treatment, which included aurora kinase B (*Aurkb*), centromere protein T (*Cenpt*), nucleolar and spindle associated protein 1 (*Nusap1*) and polo-like kinase 1 (*Plk-1*), kinetochore associated proteins and protein regulator of cytokinesis (*Prc1*) (Table 5).

**Table 5:** Transcripts encoding cell cycle regulators, kinesins, centromere, spindle and kinetochore associated proteins are upregulated by 5 mM PD169316 treatment.

Gene ID	Gene Name	Accession Number	Fold Change	p value
***cyclins*, *cyclin associated proteins*, *cyclin dependent kinase inhibitors***				
Ccnb2	cyclin B2 (Ccnb2)	NM_001009470.1	1.97	7.82E-08
*Ccna2*	cyclin A2 (Ccna2)	NM_053702.1	1.80	7.02E-08
*Ccnd1*	cyclin D1 (Ccnd1)	NM_171992.2	1.68	9.88E-07
*Cdkn1c*	cyclin-dependent kinase inhibitor 1C (P57) (Cdkn1c), transcript variant 1	NM_001033757.1	-1.85	1.71E-06
*Cdc2*	cell division cycle 2, G1 to S and G2 to M	NM_019296	2.40	1.78E-08
*Cdca1*	cell division cycle associated 1	XM_573495.1	2.15	4.74E-08
*Cdc2*	cell division cycle 20 homolog	NM_171993.1	1.51	3.25E-07
***kinesins***				
*Kifc1*	kinesin family member C1 (Kifc1)	NM_001005878.1	2.16	1.36E-07
*Kif22*	kinesin family member 22 (Kif22)	NM_001009645.1	1.81	3.54E-07
*Kif4*	kinesin family member 4 (Kif4)	XM_343797.3	1.53	8.37E-08
*Kif20a*	kinesin family member 20A (predicted)	XM_341592.3	1.52	6.71E-06
*Kif11*	kinesin family member 11 (Kif11)	XM_001060913.1	1.48	1.30E-06
*Kif23*	kinesin family member 23 (predicted)	XM_001073723.1	1.36	8.10E-06
*Kif15*	kinesin family member 15 (Kif15)	NM_181635.2	1.24	1.03E-03
***microtubules*, *microtubule networks***				
*Tubb5*	tubulin, beta 5 (Tubb5)	NM_173102.1	1.37	2.77E-05
*Tubb6*	tubulin, beta 6 (Tubb6)	NM_001025675.1	1.36	3.26E-06
*LOC498736*	similar to tubulin, beta 2 (LOC498736),	XM_574013.2	1.35	3.43E-06
*Tubg1*	tubulin, gamma 1 (Tubg1)	NM_145778.2	1.23	8.53E-05
*Tubb3*	tubulin, beta 3 (Tubb3)	NM_139254.1	1.21	3.11E-04
*Pak7*	p21 (CDKN1A)-activated kinase 7	XM_001080088.1	-1.25	7.95E-04
***actin assembly*, *vesicle transport***				
*Anln*	anillin, actin binding protein (scraps homolog, Drosophila)	XM_219687.3	1.34	8.14E-08
*Rab33a*	RAB33A, member of RAS oncogene family	XM_229145.3	-2.12	2.23E-07
***replication fork*, *DNA synthesis initiation***				
*Mcm6*	minichromosome maintenance deficient 6	XM_001055953.1	1.99	7.89E-08
*Prim1*	DNA primase, p49 subunit (Prim1)	NM_001008768.1	1.89	2.24E-07
*Rfc3*	replication factor C (activator 1) 3	NM_001009629.1	1.73	3.57E-07
*Mcm7*	minichromosome maintenance deficient 7	NM_001004203.1	1.43	9.16E-06
*Mcm2*	minichromosome maintenance deficient 2 mitotin	XM_001072364.1	1.36	3.18E-06
*Mcm3*	minichromosome maintenance deficient 3	XM_236988.4	1.35	6.18E-06
*Mcm10*	minichromosome maintenance deficient 10	XM_001071383.1	1.29	4.39E-07
***spindle formation*, *separation***				
*Aurkb*	aurora kinase B	NM_053749.1	2.09	6.10E-09
*Prc1*	protein regulator of cytokinesis 1	XM_001061201.1	2.06	9.14E-07
*Spc25*	NDC80 kinetochore complex component, homolog	NM_001009654	1.93	2.55E-07
*Spbc24*	spindle pole body component 24 homolog	XM_001077474.1	1.86	4.37E-06
*ASPM*	asp (abnormal spindle)-like, microcephaly associated (Drosophila)	XM_213891.4	1.82	2.65E-07
*Nuf2*	Kinetochore protein Nuf2	NM_001012028	1.73	4.72E-10
*Nusap1*	nucleolar and spindle associated protein 1	XM_001075591.1	1.51	1.19E-05
*Kntc1*	kinetochore associated 1	XM_001074897.1	1.43	2.48E-07
*Kntc2*	kinetochore associated 2 (predicted)	XM_001055564.1	1.37	3.13E-07
*Plk1*	polo-like kinase 1 (Drosophila) (Plk1)	NM_017100.1	1.34	6.68E-06
*Cenpt*	centromere protein T	NM_001024257	1.33	1.11E-04
***checkpoints***				
*Ttk*	Ttk protein kinase (predicted)	XM_001062174.1	1.58	2.91E-07
*Bub1*	budding uninhibited by benzimidazoles 1 homolog	XM_215849.4	1.50	1.85E-05
*Bub1b*	budding uninhibited by benzimidazoles 1 homolog, beta	XM_342494.3	1.33	1.29E-06
*Gadd45a*	growth arrest and DNA-damage-inducible 45 alpha	NM_024127.2	-1.37	2.88E-05

### p38 inhibition suspends OLPs in an active cell cycle state

**Fig 4:**
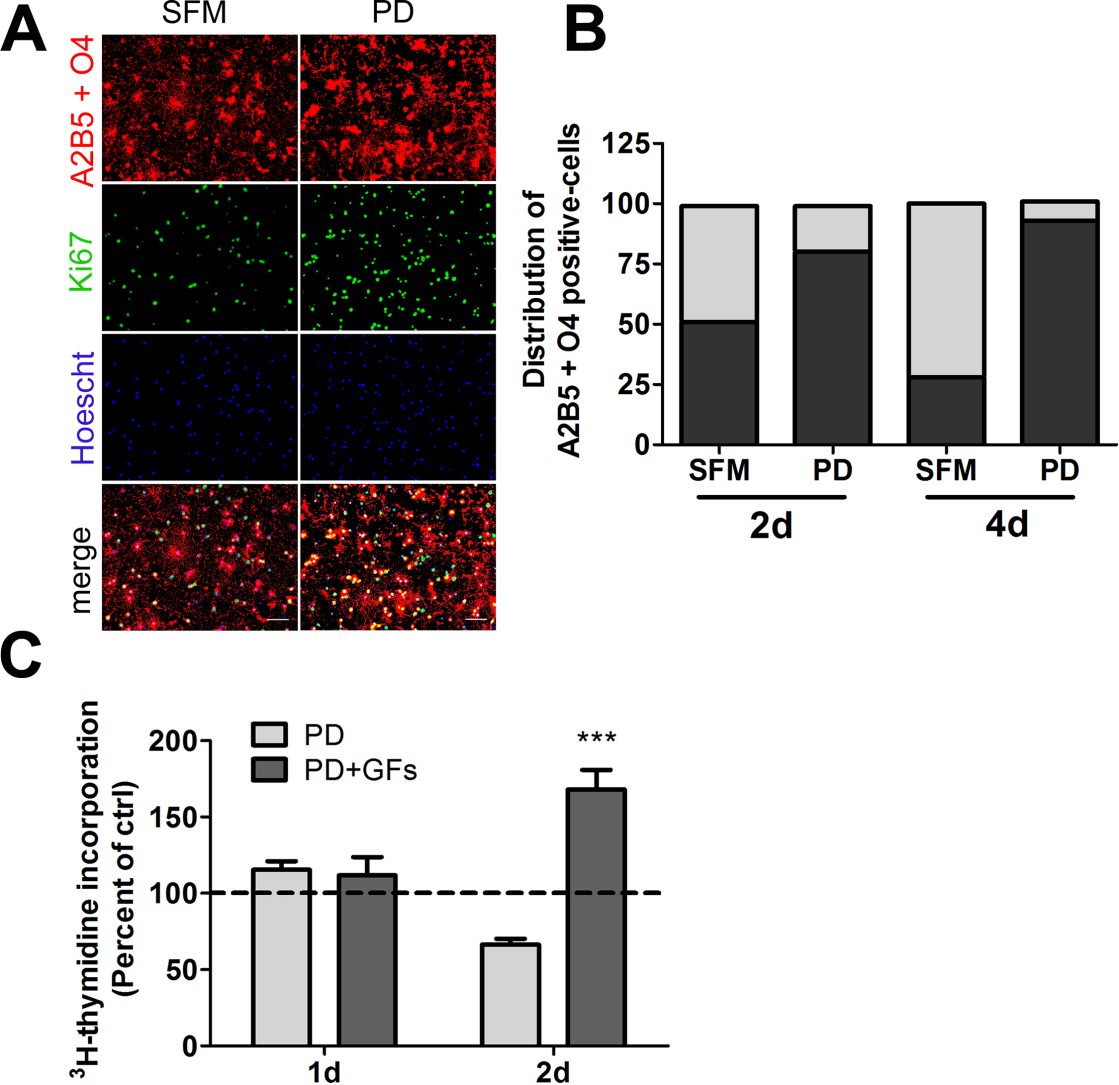
p38 inhibitors maintain oligodendrocyte progenitor cells in a non-resting state of the cell cycle. (A, B) Oligodendrocyte progenitors were maintained in serum-free medium in the presence or absence of PD169316 for 2d or 4d and the A2B5 and O4-positive population analyzed for the presence of nuclear Ki67. (A) Representative fluorescent images for the 2d time point, scale bar represents 50 μm. (B) Plot of the relative distribution of Ki67+ or Ki67- A2B5+O4-positive cells. (C) Oligodendrocyte progenitors were treated with 5 μM PD169316 for 1d or 2d, followed by ^3^H-thymidine incorporation overnight in the presence of absence of growth factors (PDGF-AA and bFGF). Statistical differences in (C) were determined by one-way ANOVA followed by Dunnett’s correction (***p< 0.001 vs ctrl).

Given the decreased capacity of PD169316-treated cells to incorporate thymidine, we further explored the cell cycle state of p38-inhibited OLPs over a longer time frame by evaluating the presence of nuclear protein Ki67 in cells double-labeled with monoclonal antibodies to OLP markers: A2B5 and O4, as cells positive for either marker can proliferate. Ki67 is expressed during all active phases of the cell cycle with the exception of G0 resting stage. OLPs were treated with either PD169316 or DMSO control for 2 or 4d. The populations of Ki67-positive cells and A2B5- and O4-labeled cells were enumerated for each group and compared (Fig 4A and 4B). While fewer than 30% of A2B5 and O4 positive cells treated in DMSO control were Ki67-positive, the significant majority of PD169316-treated A2B5- or O4-positive cells were labeled by the Ki67 marker at 4d. The trend was similar at the 2d time point but the difference in the number of Ki67 labeled cells was smaller. Application of mitogens at the 4d time point to treatment and control cells had no significant effect on the proportion of Ki67 labeled cells over those observed in DMSO controls (data not shown). Our results suggest that p38 inhibition over a prolonged duration suspends cells in a non-resting cell state and prevents their differentiation, thus providing a potential mechanism for the function of p38 in oligodendrocyte differentiation and myelination.

### p38 inhibition blocks OLG differentiation, while maintaining OLPs in a proliferation competent state

Given that our microarray analyses detected an increase in the expression of genes involved in cell cycle and mitosis after p38 inhibition, with accompanying decreases in transcripts encoding CDKIs and the thyroid hormone receptor, we explored whether PD169316 treatment perturbs cell cycle processes in OLPs. ^3^H -thymidine incorporation was performed on cells treated with PD169316 for 1, and 2d (Fig 4). Notably, ^3^H-thymidine incorporation decreased over controls at the 2d time point (Fig 4C). However, when PDGF-AA and bFGF mitogens were applied to PD169316-treated cells for 1d more, a significant increase in thymidine incorporation was observed suggesting that a greater proportion of treated OLPs had the capacity to enter an S-phase cell cycle proliferative state compared to OLPs treated with SFM alone (Fig 4C and S1 Fig). These results combined with the Ki67 assay suggest that p38 may play a role in controlling cell cycle progression in OLPs.

## Discussion

We previously reported that p38 MAPK is required for OLG differentiation and myelination of cultured dorsal root ganglia neurons. To better understand how p38 regulates OLG differentiation, we used a rat whole genome microarray analysis to globally survey gene expression changes in primary OLGs treated with the p38 inhibitor PD169316. Grouping the genes into functional classes using either UniProt or literature searches highlighted an upregulation of groups of genes involved in cytokinesis, centromere and spindle formation, replication and cell cycle progression. Smaller subsets of genes upregulated by PD169316 treatment included amino acid transporters, cytoskeletal proteins, vesicle transporters, extracellular matrix molecules, and genes involved in oxidative stress. In contrast, the most prominent group of down-regulated genes was generally unclassified or had no known function. Among other downregulated genes were bone morphogenetic proteins, chondroitin sulfate proteoglycans, and cytoskeletal/vesicle trafficking proteins.

In agreement with other studies, p38 inhibition upregulated myelin-specific gene products associated with OLG differentiation [22, 23]. In alignment with our previous study, p38 inhibition downregulated a large number of myelin specific transcripts, including *Mag*, *Mbp*, *Cnp*, *Plp* and *Mobp* [5]. Novel p38 pathway targets identified in our microarray analysis included the lipid synthesis gene CGT, and squalene epoxidase, a cholesterol biosynthesis enzyme [24]. We also observed decreases in the mRNA levels of pro-myelin gene activators including HDAC11, Fyn and Zfp488. HDAC11 is responsible for activating MBP and PLP promoters, and its knockdown decreases protein levels [12]. The tyrosine kinase Fyn plays multiple roles in oligodendrocyte differentiation, including activation of MBP gene promoter, transport of MBP mRNAs, and phosphorylation of MAG [25, 26]. Zfp488 is expressed in differentiating OLGs [27] and it was found to be transcriptionally downregulated in OLG-specific p38α knockout mice at neonatal stages. Furthermore, p38α knockout delays OLP differentiation and causes a number of myelin ultrastructural abnormalities, in addition to a reduction in the number and diameter of axons [8]. Interestingly, our previous results in DRGN/OLG cocultures treated with p38 inhibitors showed a complete and irreversible inhibition of myelination and a lack of organization of the axo-glial adhesion molecule Caspr, while the inhibition of differentiation in OLP cultured alone was reversible [5] suggesting that p38 may also play a role in axonal integrity.

Another group of gene transcripts that were upregulated in OLPs treated with PD169316 included the upstream enzymes regulating p38 activity such as MKKKs (*Dlk1*), and PBK, a MKK that specifically phosphorylates p38 during cell cycle [17, 28]. These results are in agreement with genetic ablation studies of p38α in cardiomyocytes which augmented the expression of p38 upstream activators [29] and suggest that these are compensatory mechanisms in a cellular attempt to re-establish basal p38 levels.

Treatment with p38 inhibitor resulted in up-regulation of inhibitors of OLG differentiation or transcriptional repressors: *Id1*, *Id2*, *Tcf4*, *and Sox6* [16, 30–32] as well as *Nestin*, a marker of immature OLG [33]. In contrast, Nkx2.2, which normally decreases with OLG differentiation was increased by PD169316 treatment [34]. However, the p38 pathway appears to differentially regulate immature cell-stage gene targets that are normally downregulated during OLG lineage progression, since no upregulation of *Pdgfra* was found with p38 inhibitors. Furthermore, reduced levels of thyroid hormone receptor alpha (*Thra*) transcripts suggest that p38 inhibition may disrupt the intracellular timer that regulates OLG differentiation.

The largest subset of upregulated genes induced by PD169316 treatment was associated with cell cycle, chromosome spindle formation, cytokinesis, centromere formation, kinesin molecular motors, and microtubule/microfilament organization. Many of these genes are involved in mitosis, and spindle formation (reviewed by [35]); and decrease with OLG differentiation [22]. In addition, we also detected gene expression increases in cell cycle regulators including *Cyclin A2*, *B1*, *D1*, and the aurora kinases suggesting that treatment of OLPs with p38 inhibitors increased cellular proliferation. In contrast, expression of cyclin dependent kinase inhibitors, *p57* ^*kip2*^ and *p21* ^*cip1*^ were decreased. Both molecules are important for OLP differentiation by regulating cell cycle state transitions and timing of OLP differentiation [36, 37]. In other cell types, p38 is involved in the regulation of DNA checkpoints including the G2/M, G1/S and G1/G0 [21]. Examination of Ki67-positive OLPs revealed that the majority of p38 inhibited cells were suspended in an active phase of the cell cycle. Notably, addition of PDGF-AA and bFGF allowed some OLPs to re-enter S-phase of the cell cycle as shown by thymidine incorporation. These results are in agreement with those obtained in cardiomyocytes [38–40], and mouse embryonic fibroblasts [41] showing that p38 MAPK pathway regulates genes involved in cellular response to stress, cell division, cell signaling, inflammation and adhesion.

Although the mechanisms by which p38 pathway regulates the cell cycle in OLPs remains to be fully elucidated, our data provides strong support for its role in promoting a transition state between cell proliferation and differentiation through its transcriptional repression of genes involved in cell cycle and inhibitors of differentiation while promoting the expression of pro-myelinating factors. Further studies will be necessary to fully reveal the specific molecular components involved in this regulatory process. Our microarray dataset will serve as a useful resource for future studies investigating the mechanisms by which p38 regulates oligodendrocyte proliferation, differentiation and myelination.

## Supporting Information

S1 Fig p38 inhibitors maintain oligodendrocyte progenitor cells in a non-resting state of the cell cycle. OLPs treated for 1d or 2d (A, C) with PD169316 decreased ^3^H-thymidine incorporation. However, when cultures are maintained for 1d or 2d with PD169316 and then stimulated with PDGF-AA and bFGF for an additional 1d, some PD-treated OLPs have the capacity to incorporate thymidine (B, D). Statistical differences were determined by one-way ANOVA followed by Dunnett’s correction (*p < 0.05, **p< 0.01, ***p< 0.001 vs ctrl).
(TIF)

S1 Table List of genes upregulated an downregulated in OLPs following 1d of treatment with the p38 inhibitor PD169316. (XLSX)

S2 Table qPCR primers for gene validations. (DOCX)

## Abbreviations

CDKI: cyclin dependent kinase inhibitor
CGT–UDP: galactose ceramide galactosyltransferase
CNP: 2’, 3’-cyclic nucleotide 3’-phosphodiesterase
CNS: central nervous system
GalC: galactosylcerebroside / galactosylceramide
HDAC: histone deacetylase
Id2: inhibitor of differentiation 2
MAG: myelin-associated glycoprotein
MBP: myelin basic protein
MAPK: mitogen-activated protein kinase
MK2: MAPK activated protein kinase 2
MKK: MAPK kinase
MKKK: MKK kinase
MOG: myelin oligodendrocyte glycoprotein
OLIG2: oligodendrocyte transcription factor 2
OLG: oligodendrocyte
OLP: oligodendrocyte progenitor
PBK: PDZ domain binding kinase
qRT-PCR: quantitative real-time PCR
SFM: serum free media
Tcf4: transcription factor 4
