# Supplementary figures and images for: p38 Mitogen-Activated Protein Kinase Pathway Regulates Genes during Proliferation and Differentiation in Oligodendrocytes
Source: PLoS One. 2015 Dec 29;10(12):e0145843. doi: 10.1371/journal.pone.0145843 (PMC4699908; doi:10.1371/journal.pone.0145843)

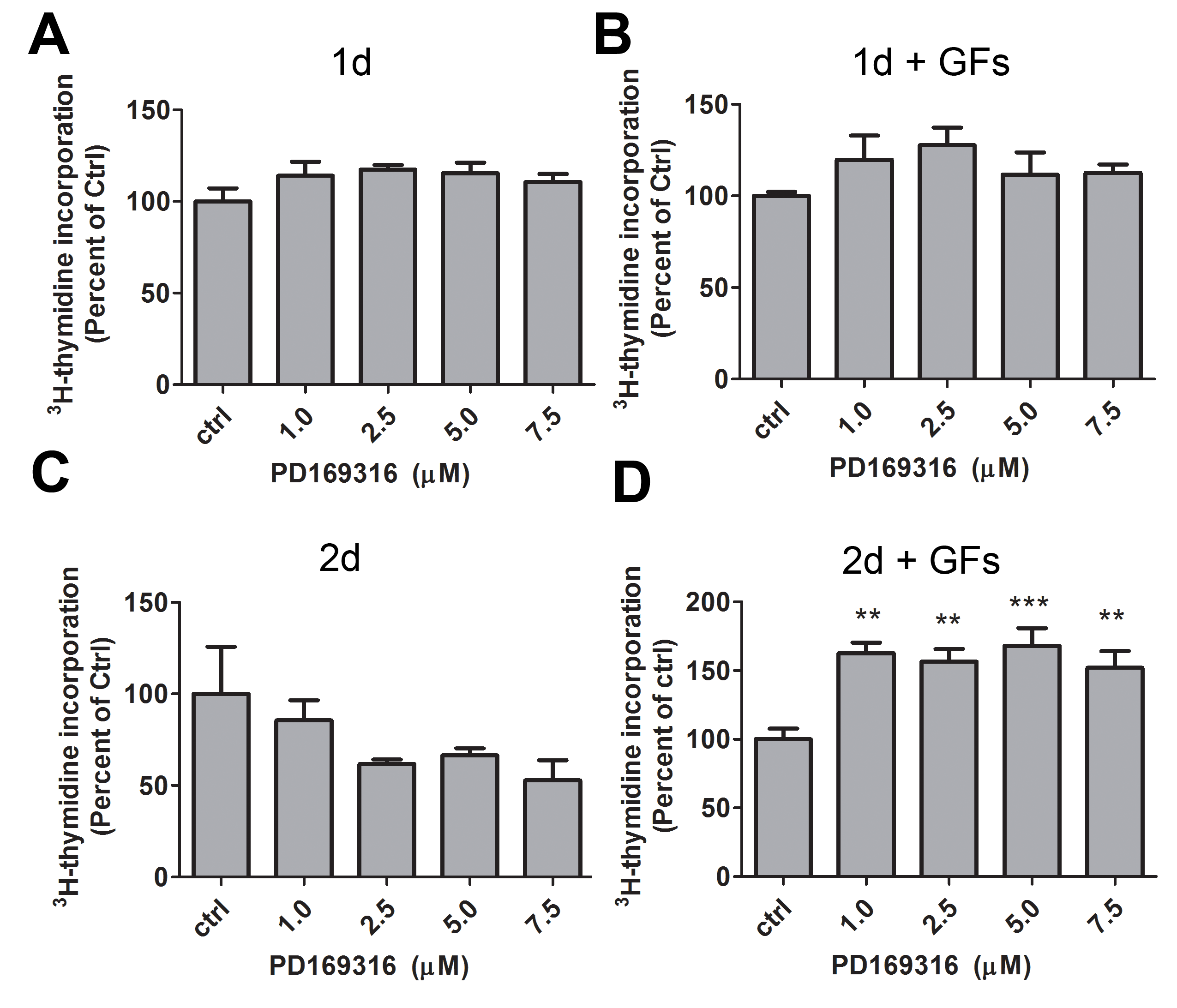

Supplement: S1 Fig — OLPs treated for 1d or 2d (A, C) with PD169316 decreased 3H-thymidine incorporation. However, when cultures are maintained for 1d or 2d with PD169316 and then stimulated with PDGF-AA and bFGF for an additional 1d, some PD-treated OLPs have the capacity to incorporate thymidine (B, D). Statistical differences were determined by one-way ANOVA followed by Dunnett’s correction (*p < 0.05, **p< 0.01, ***p< 0.001 vs ctrl). (TIF) [file pone.0145843.s001.tif]
