# Supplementary material for: p38 Mitogen-Activated Protein Kinase Pathway Regulates Genes during Proliferation and Differentiation in Oligodendrocytes
Source: PLoS One. 2015 Dec 29;10(12):e0145843. doi: 10.1371/journal.pone.0145843 (PMC4699908; doi:10.1371/journal.pone.0145843)
Supplement: S2 Table — (DOCX) [file pone.0145843.s003.docx]

**Supplemental Table 2: qPCR primers for gene validations**

| **Gene Name** | **Accession** | **Forward Primer 5’→ 3’** | **Reverse Primer 5’ → 3’** |
| --- | --- | --- | --- |
| *Mag* | NM_017190.4 | TCAACAGTCCCTACCCCAAG | GAGAAGCAGGGTGCAGTTTC |
| *Fyn* | NM_012755.1 | GCAGCTTGTACAGCATTACTCAG | TCCCTTTGTGACAGGGAACT |
| *Hdac11* | NM_001106610.2 | AGCTGGCTGTGGAACGAG | CACTGGAGCAGTGGTGGA |
| *Tcf4* | NM_053369.1 | GGAGGAGAAGAACTCGGAAAACT | CATTGACCAACGACGACTTGA |
| *Hes5* | NM_024383.1 | ATGCTCAGTCCCAAGGAGAA | CAGTTTCAGCTGCTCAATGC |
| *Id2* | NM_013060.3 | CGGAACACCACACTGATGAC | GGACAGAACCAAACGTCCAG |
| *Nkx2.2* | NM_001191904.1 |  |  |
| *Rab33a* | NM_001108257.1 | GCCACTATCGGTGTGGACTT | TCTTGTCCTGCCGTGTCC |
| *p57kip2* | AJ488291.1 | CGAGGAGCAGGACGAGAA | GCTTGGCGAAGAAGTCGT |
| *p21Cip1* | BC100620.1 | GACATCTCAGGGCCGAAA | GGCGCTTGGAGTGATAGAAA |
| *Cyclin A1* | NM_001011949.1 | GAGGGAAATTGCAGCTTGTC | GGGCGGGTATATCTCTTCGT |
| *Cyclin D1* | D14014.1 | AGCAGAAGTGCGAAGAGGAG | GGCTCCAGAGACAAGAAACG |
